# The impact of transcranial direct current stimulation on brain network connectivity and topology in post-stroke cognitive impairment patients: a resting-state fMRI study

**DOI:** 10.1007/s10072-025-08348-8

**Published:** 2025-07-12

**Authors:** Jiali Zhong, Xiaoshan Jing, Ying Liang, Pan Hao, Ruchen Peng, Ruiqiang Xin

**Affiliations:** 1 Department of Radiology, Beijing Luhe Hospital, Capital Medical University, Beijing, 101149 China; 2 Department of Rehabilitation, Beijing Luhe Hospital, Capital Medical University, Beijing, 101149 China; 3 School of Biomedical Engineering, Capital Medical University, Beijing, 100069 China

**Keywords:** Transcranial direct current stimulation, Post-stroke cognitive impairment, Resting-state fMRI, Functional connectivity, Graph theory, Cognitive function

## Abstract

**Background:**

Post-stroke cognitive impairment (PSCI) is a common and severe consequence of ischemic stroke (IS) that significantly affects patient outcomes. Transcranial direct current stimulation (tDCS) has shown promise in enhancing cognitive function in IS patients, but its underlying mechanisms are not fully understood. This study investigates the effects of tDCS on brain functional connectivity and network topology using resting-state functional magnetic resonance imaging (rs-fMRI).

**Methods:**

In this double-blind study, sixty-five IS patients with PSCI were randomly assigned to either the tDCS or control group. Rs-fMRI data were acquired before and after the intervention. We analyzed functional connectivity (FC) and graph theory-based topological properties. Cognitive performance was assessed using the Mini-Mental State Examination (MMSE) and Montreal Cognitive Assessment (MoCA).

**Results after:**

treatment, both groups showed improvements in MMSE and MoCA scores, with the tDCS group demonstrating significantly greater improvements (*p* < 0.05). In the tDCS group, FC significantly increased between four pairs of brain regions (*p* < 0.05, FDR-corrected). Additionally, Global Efficiency (*E*_*g*_) significantly improved (*p* < 0.05, FDR-corrected), and this improvement positively correlated with enhancements in MMSE scores (*r* = 0.403, *p* = 0.037).

**Conclusion:**

These findings suggest that tDCS improves cognitive function in PSCI by altering brain network connectivity and topological organization, providing neuroimaging evidence to support its therapeutic mechanisms.

**Supplementary Information:**

The online version contains supplementary material available at 10.1007/s10072-025-08348-8.

## Introduction

The global incidence of stroke has risen due to aging populations and increasing cardiovascular risk factors, with ischemic stroke (IS) accounting for approximately 80% of cases [1–3]. Many patients miss the critical window for acute reperfusion therapies, and survivors often suffer from significant neurological deficits, requiring extensive support. As a result, stroke remains the leading cause of disability worldwide [4–6]. Post-stroke cognitive impairment (PSCI) is a common complication that often leads to cognitive deficits, behavioral changes, and dementia, significantly reducing the quality of life [7–10]. Effective and timely rehabilitation is therefore essential for cognitive recovery in IS patients.

In addition to conventional approaches such as cognitive training and pharmacotherapy, transcranial direct current stimulation (tDCS), a non-invasive neuromodulation technique, has been increasingly used in clinical cognitive rehabilitation. Research indicates that tDCS improves cognitive domains in IS patients, including attention, memory, and language expression [11–13]. However, the neural mechanisms underlying these improvements, especially regarding brain network dynamics, are not well understood due to limited neuroimaging evidence.

Resting-state functional magnetic resonance imaging (rs-fMRI) uses blood-oxygen-level-dependent (BOLD) signals to assess brain function, capturing hemodynamic changes that reflect underlying neurophysiological activity [14–17]. Previous studies have shown that tDCS alters rs-fMRI metrics, such as fractional amplitude of low-frequency fluctuation (fALFF) and regional homogeneity (ReHo), in patients with mild cognitive impairment, linking these changes to cognitive gains [18, 19]. However, these studies often focus on localized brain activity, neglecting the brain’s integrated functionality, which depends on coordinated interactions between regions [20]. Notably, even localized IS lesions can disrupt global functional connectivity via neuroplasticity, suggesting that tDCS may facilitate PSCI recovery by reorganizing whole-brain networks. However, this hypothesis requires further investigation.

Graph theory provides a robust framework for analyzing brain networks, representing regions as nodes and their connections as edges to quantify topological properties such as efficiency and clustering [21–23]. This approach has revealed functional reorganization following central nervous system injuries, offering novel insights into tDCS-induced changes [24]. Using rs-fMRI, this study investigates how tDCS affects brain functional connectivity and network topology in PSCI patients. The goal is to provide neuroimaging evidence that clarifies the cognitive benefits of tDCS.

## Materials and methods

### Subjects

Sixty-five IS patients with PSCI were recruited from the Department of Neurology at Beijing Luhe Hospital, Capital Medical University, between October 2021 and November 2024. A rehabilitation physician with eight years of experience confirmed cognitive impairment using the Mini-Mental State Examination (MMSE) and Montreal Cognitive Assessment (MoCA). The study adhered to the Declaration of Helsinki and was approved by the Medical Ethics Committee of Beijing Luhe Hospital (Approval No. 2022-LHKY-007-01). All participants provided written informed consent.

### Inclusion criteria

- Aged 35–75 years, right-handed.
- First IS occurrence within 14 days.
- Unilateral basal ganglia lesions (0.3–1.5 cm).
- Capable of completing MRI and cognitive assessments.
- No pre-existing cognitive decline.

### Exclusion criteria

- Severe intracranial lesions beyond IS.
- MRI contraindications (e.g., claustrophobia, metal implants).
- Unstable vital signs.

After acute-phase treatment, patients were randomized into tDCS (*n* = 33) and control (*n* = 32) groups using a computer-generated random number sequence. The study used a double-blind design, with both participants and outcome evaluators blinded to group assignments. Group assignments were concealed in sealed envelopes until data collection was complete. Both groups received standard pharmacological treatments, including antiplatelet agents (e.g., Aspirin, Clopidogrel bisulfate tablets), neuroprotective and repair agents (e.g., Edaravone), and cerebral metabolic improvers (e.g., Citicoline). The tDCS group received active transcranial direct current stimulation, while the control group received sham stimulation. Due to early discharge, five patients were excluded, and seven were excluded because of poor rs-fMRI quality. This resulted in final samples of 27 patients in the tDCS group and 26 in the control group. Demographic characteristics (gender, age, education), clinical variables including time from stroke onset, lesion laterality (left/right), and modifiable risk factors (hypertension, diabetes) were systematically recorded.

### MRI data acquisition

MRI data were obtained using a 3.0 Tesla scanner (uMR 780; UIH, Shanghai, China) equipped with a 24-channel head coil. Participants were positioned supine and instructed to keep their eyes closed, stay awake, and avoid active thinking during the scanning process.

T1-Weighted 3D-FSP Sequences:

- Field of view (FOV): 256 mm × 256 mm.
- Repetition time (TR): 6.9 ms, Echo time (TE): 3.0 ms, Inversion time: 750 ms.
- Slice thickness: 1.0 mm, 176 slices.
- Flip angle: 9°, Matrix: 256 × 256.

Rs-fMRI Sequences:

- FOV: 230 mm × 230 mm.
- TR: 2000 ms, TE: 30 ms.
- Slice thickness: 3.5 mm, 39 slices.
- Flip angle: 90°, Matrix: 102 × 102.

### Rs-fMRI data preprocessing

We performed data preprocessing using DPARSFA [25], which is part of DPABI [26], along with SPM 12, all based on MATLAB 2023a. The workflow included:

1. Conversion from DICOM to NIfTI format.
2. Removal of the initial 10 time points.
3. Slice timing correction.
4. Head-motion correction (exclusion threshold: >3 mm displacement or > 3° rotation).
5. Spatial normalization to Montreal Neurological Institute (MNI) space (voxel size: 3 mm × 3 mm × 3 mm).
6. Detrending to eliminate non-neural trends.
7. Regression of signals from white matter and cerebrospinal fluid, and 24 head-motion parameters.

### Functional connectivity analysis

Functional connectivity (FC) was evaluated using the GRETNA toolbox [27]. Preprocessed BOLD data were bandpass-filtered (0.01–0.08 Hz), and the AAL-90 atlas segmented the cortex into 90 regions. We calculated Pearson correlation coefficients of BOLD signals between each pair of regions across 230 time points, resulting in a 90 × 90 correlation matrix. This matrix was then Fisher-Z transformed for normalization.

### Topological analysis of the functional network

Network topology was evaluated using GRETNA, applying sparsity thresholds from 0.05 to 0.40 (step = 0.02). Correlation matrices were binarized (1 = connection, 0 = no connection), and global properties were calculated:

- Global Efficiency (*E*_*g*_): Mean efficiency of information transfer across nodes.
- Small-World Property (*σ*): Measure of small-world network attributes.

Area under the curve (AUC) values were derived for each metric across the sparsity range.

### Statistical analysis

Statistical analyses were performed using SPSS 27.0. Categorical variables (including gender, lesion laterality [left/right], hypertension, and diabetes) were assessed with chi-square tests. Continuous variables (age, education, time from stroke onset, and cognitive scores) were evaluated using two-independent-samples *t*-tests or Mann-Whitney *U* tests based on distributional assumptions and variance equality verification. Paired *t*-tests examined before and after treatment changes in topological properties within groups (FDR-corrected, *p* < 0.05). Pearson correlations explored associations between significant brain metrics and cognitive scores.

## Results

### Demographics and clinical profiles

There were no significant differences between the tDCS and control groups in terms of age, gender, education, onset time, lesion laterality (left/right), or modifiable risk factors like hypertension and diabetes (Table 1).

**Table 1:** Participant Demographics and Clinical Profiles

	tDCS group	control group	*χ*^*2*^ / *Z*-value	*p*-value
**Demographics**				
Gender, n (M/F)	27 (18/9)	26 (16/10)	0.135	0.714
Age, years	67.96 ± 4.35	65.23 ± 5.28	-1.827	0.068
Education, years	7.07 ± 1.21	6.80 ± 1.44	-0.825	0.409
**Stroke Characteristics**				
Onset time, days	10.37 ± 1.02	9.84 ± 1.23	-1.551	0.121
Lesion laterality (Left/Right)	27 (17/10)	26 (14/12)	0.453	0.501
**Vascular Risk Factors**				
Hypertension (Yes/No)	27 (21/6)	26 (19/7)	0.158	0.691
Diabetes (Yes/No)	27 (9/18)	26 (6/20)	0.687	0.407

### Cognitive scale scores

After treatment, both groups showed significant improvements in MMSE and MoCA scores (*p* < 0.001). The tDCS group exhibited significantly greater improvements compared to the control group (*p* < 0.001) (Table 2).

**Table 2:** Differences in MMSE and MoCA Scores Between Groups

Scale	Group	Before treatment	After treatment	Difference	Between-Group *t*-value	*p*-value
**MMSE**						
	tDCS	20.37 ± 1.44	24.44 ± 1.71	4.07 ± 1.33	-2.332	0.009*
	Control	20.46 ± 1.45	23.62 ± 2.09	3.15 ± 1.17		
**MoCA**						
	tDCS	19.78 ± 2.06	24.30 ± 2.20	4.51 ± 1.29	-2.629	0.020*
	Control	19.46 ± 1.62	22.85 ± 1.92	3.38 ± 1.44		

*Note: *p < 0.05, MMSE: Mini-Mental State Examination, MoCA: Montreal Cognitive Assessment

### Functional connectivity

**Fig. 1:**
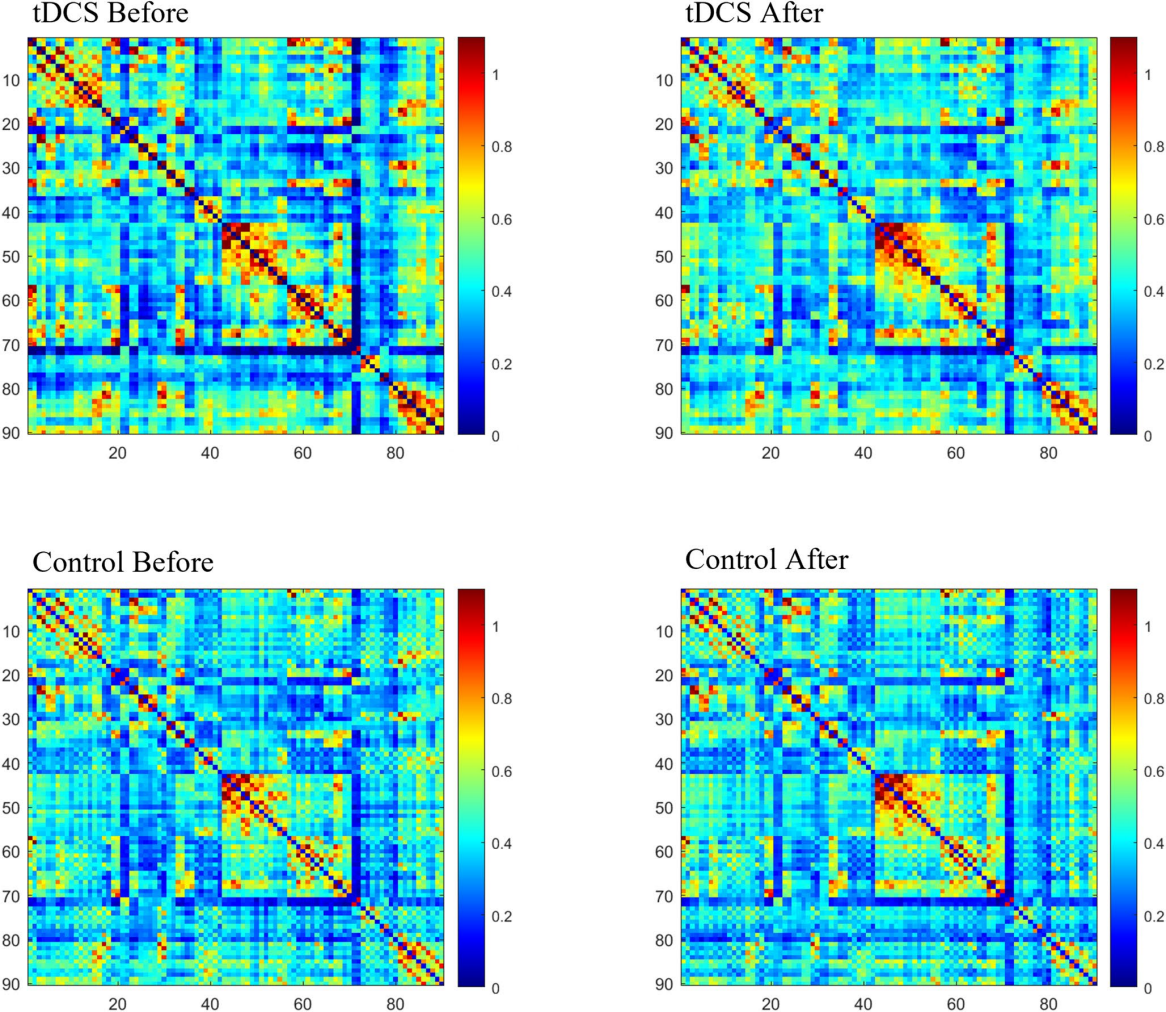
Group-level resting-state FC maps before and after treatment for tDCS and control groups using the AAL-90 atlas. Fisher-Z-transformed FC coefficients are visualized (blue: *Z* < 0, red: *Z* > 0)

**Fig. 2:**
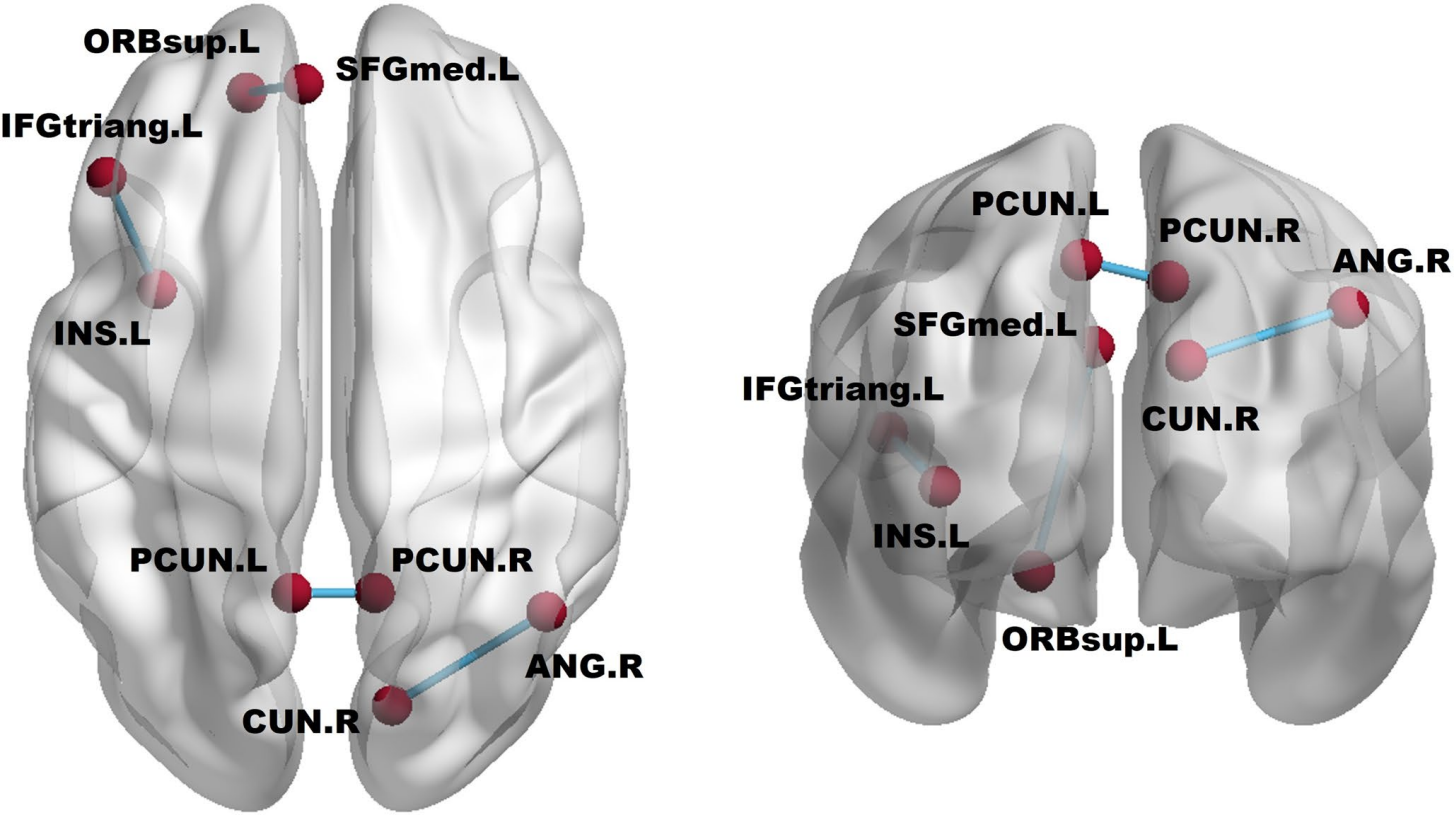
Significant FC increases in the tDCS group: CUN.R–ANG.R, PCUN.L–PCUN.R, IFGtriang.L–INS.L, ORBsup.L–SFGmed.L (*p* < 0.05, FDR-corrected)

After treatment, the tDCS group showed significantly increased FC in four region pairs: right cuneus (CUN.R) with right angular gyrus (ANG.R), left precuneus (PCUN.L) with right precuneus (PCUN.R), left triangular part of inferior frontal gyrus (IFGtriang.L) with left insula (INS.L), and left orbital part of superior frontal gyrus (ORBsup.L) with left medial superior frontal gyrus (SFGmed.L) (*p* < 0.05, FDR-corrected). No significant changes were observed in the control group (Figs. 1 and 2).

### Functional network topological properties

**Fig. 3:**
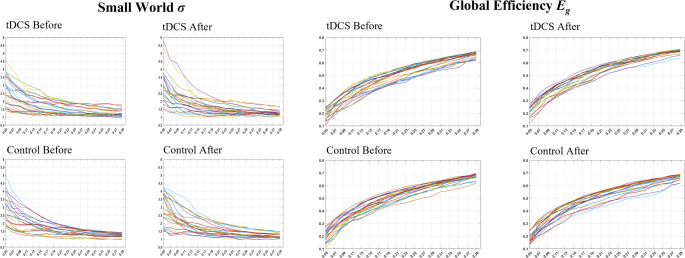
Fitting curves of *σ* and *E*_*g*_ across sparsity thresholds (0.05–0.40, step = 0.02) for both groups before and after treatment. *σ*: Small-World Property, *E*_*g*_: Global Efficiency

**Fig. 4:**
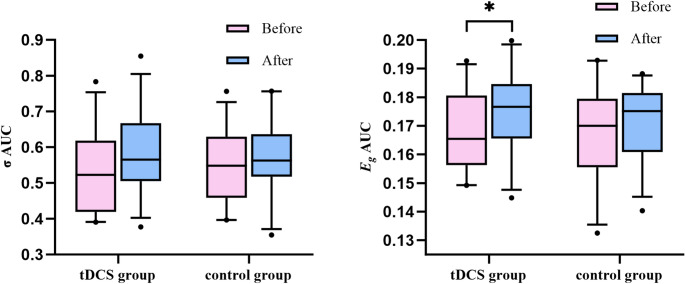
AUC differences in *E*_*g*_ and *σ* before and after treatment (*p* < 0.05, FDR-corrected). *Note: *p < 0.05, AUC: area under the curve, *σ*: Small-World Property, *E*_*g*_: Global Efficiency

In the tDCS group, there was a significant increase in *E*_*g*_ after treatment (*p* < 0.05, FDR-corrected), while *σ* did not change significantly. The control group exhibited no significant alterations in either metric (Table 3; Figs. 3 and 4).

**Table 3:** Differences in AUC of σ and Eg

	AUC Before	AUC After	*p*-value	*t*-value
***σ***				
tDCS group	0.5345 ± 0.1103	0.5789 ± 0.1041	0.064	-1.85
Control group	0.5634 ± 0.0993	0.5483 ± 0.1068	0.603	-0.521
***E***_***g***_				
tDCS group	0.1684 ± 0.0129	0.1763 ± 0.0127	0.021*	-2.306
Control group	0.1677 ± 0.0164	0.1701 ± 0.0132	0.515	-0.658

*Note: *p < 0.05, AUC: area under the curve, σ: Small-World Property, Eg: Global Efficiency

### Correlation analysis

**Fig. 5:**
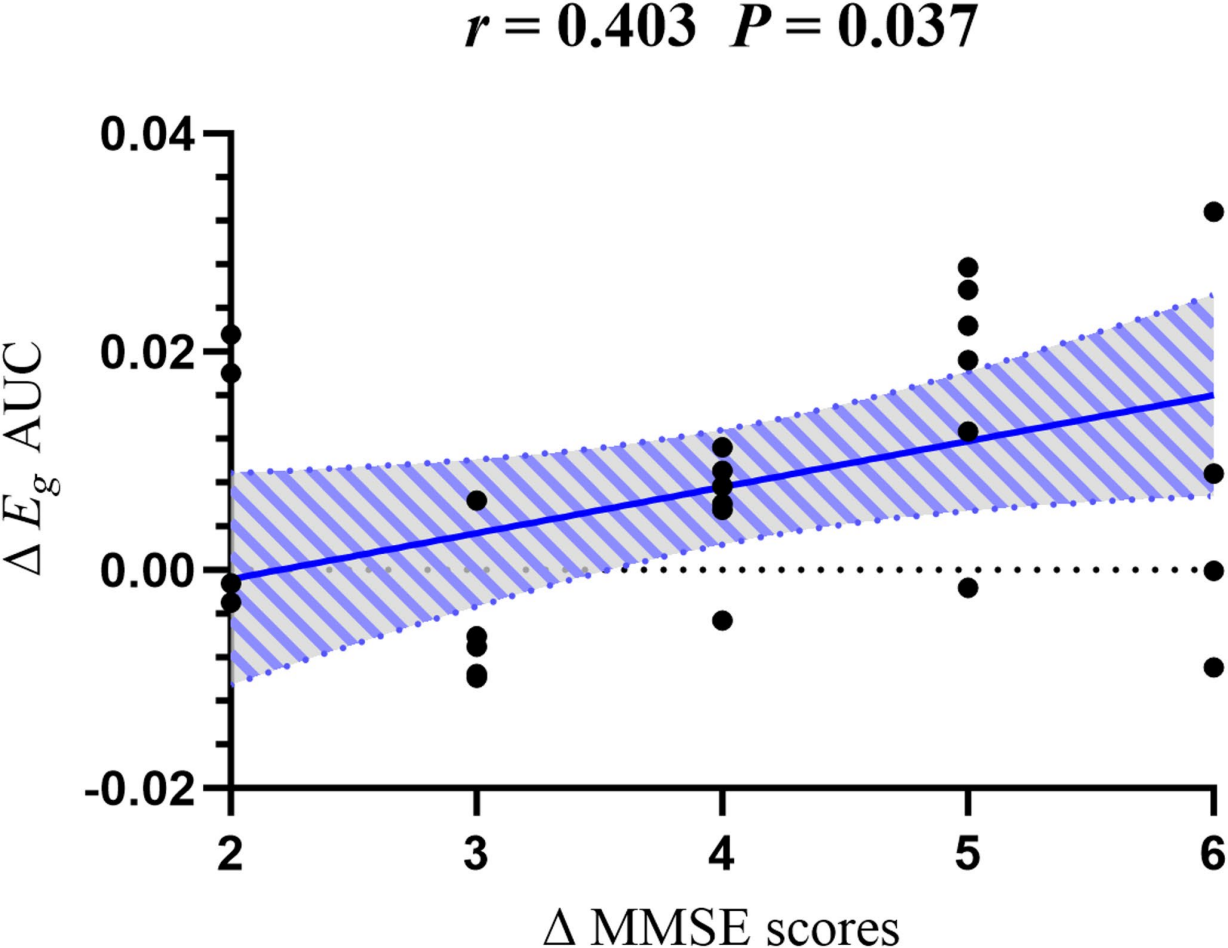
Correlation between Δ*E*_*g*_ AUC and ΔMMSE in the tDCS group (*r* = 0.403, *p* = 0.037). *E*_*g*_: Global Efficiency, MMSE: Mini-Mental State Examination

In the tDCS group, the increase in *E*_*g*_ positively correlated with improvements in MMSE scores (*r* = 0.403, *p* = 0.037) (Fig. 5). No other significant correlations were identified.

## Discussion

This study elucidates the effects of tDCS on brain functional connectivity and network topology in PSCI patients, providing insights into its mechanisms of cognitive enhancement. After treatment, both groups demonstrated significant improvements in MMSE and MoCA scores, but the tDCS group showed superior outcomes (*p* < 0.05). Additionally, the tDCS group exhibited increased FC in specific region pairs and increased *E*_*g*_, which correlated with cognitive improvements, offering robust neuroimaging evidence of tDCS’s therapeutic efficacy.

The cognitive gains in both groups likely stem from conventional therapies, such as pharmacotherapy and cognitive rehabilitation, consistent with prior studies [28–31]. However, the tDCS group experienced greater improvements, which may be attributed to the additive effect of anodal stimulation on the dorsolateral prefrontal cortex (DLPFC). This anodal stimulation may depolarize neuronal membranes, thereby increasing excitability and promoting cognitive recovery [32, 33]. This mechanism is supported by studies showing that bilateral DLPFC tDCS enhances cognition in post-stroke patients [13]. Recent studies further confirm tDCS’s cognitive benefits, with trials reporting sustained improvements in attention and working memory [11] and systematic reviews highlighting its efficacy across multiple cognitive domains [13].

FC analysis quantifies the temporal correlation of signals from different brain regions, revealing the dynamic patterns of functional collaboration in the brain. It serves as a crucial bridge linking neural activities with cognitive behaviors [34]. In neurorehabilitation, FC analysis helps demonstrate how interventions like tDCS affect the brain’s functional network, which is essential for understanding mechanisms and evaluating efficacy [35]. This study revealed tDCS-induced enhancements in regions linked to the prefrontal cortex and default mode network (DMN), critical for working memory, attention, and self-awareness [36, 37]. Specifically, the increased FC in the precuneus, a key hub of the default mode network (DMN), may indicate restored network integrity post-stroke, contributing to cognitive improvements [38]. This aligns with prior findings of disrupted DMN and frontoparietal network connectivity in PSCI, correlated with cognitive deficits [10], and extends these observations by showing tDCS’s capacity to enhance these networks. A study also noted altered static and dynamic FC in PSCI, suggesting tDCS addresses dynamic network impairments [39].

The human brain acts as a complex system achieving cognition through local functional connectivity and global integration of neurons. Thus, although ischemic stroke (IS) causes focal damage, it may disrupt functional/structural connectivity in remote brain regions, impacting cognition [40]. Graph theoretical analysis reveals the global organizational patterns of information processing in the brain by quantifying the topological properties of brain functional networks [41]. Graph theory analysis revealed that tDCS significantly increased *E*_*g*_, indicating enhanced global information transfer efficiency. This improvement correlated with MMSE score gains (*r* = 0.403, *p* = 0.037). ​These findings align with reports of reduced *E*_*g*_ in stroke patients [42] and studies linking rehabilitation success to increased *E*_*g*_ [24]. However, no similar association was observed with MoCA scores. The absence of *E*_*g*_ -MoCA correlation may reflect MoCA’s assessment of higher-order cognitive domains (e.g., executive function and complex reasoning) that could require longer interventions to manifest efficiency-related neural changes. Thus, tDCS likely facilitates cognitive recovery by optimizing brain network efficiency. The lack of change in *σ* further suggests selective enhancement of efficiency-related mechanisms rather than comprehensive network reorganization.

This study has several limitations: a modest sample size (*n* = 53 after exclusions), a focus on unilateral basal ganglia lesions, and a short-term intervention duration. These factors may limit the generalizability of the findings and restrict insights to short-term effects. The short-term nature of the study was due to the constraints of hospital stay duration, which limited the treatment and observation period. Future research should explore the long-term effects of tDCS on cognitive function and brain network changes, potentially incorporating multi-modal imaging techniques such as diffusion tensor imaging (DTI) to examine whether improvements in functional connectivity translate to structural connectivity changes over time.

## Conclusion

This investigation confirms that tDCS enhances cognitive function in PSCI by modulating brain functional connectivity and elevating global network efficiency. These findings support tDCS as a valuable adjunct in stroke rehabilitation by yielding neuroimaging evidence of its effects on brain networks. They also underscore the importance of network-based approaches in understanding cognitive recovery mechanisms.

## Electronic supplementary material

Below is the link to the electronic supplementary material.

Supplementary Material 1

Supplementary Material 2

## Data Availability

The data used in the present study can be provided upon reasonable request.
